# Supplementary material for: Financial vulnerability and the impact of COVID-19 on American households
Source: PLoS One. 2022 Jan 14;17(1):e0262301. doi: 10.1371/journal.pone.0262301 (PMC8759691; doi:10.1371/journal.pone.0262301)
Supplement: S1 File — Survey Questionnaire. (DOCX) [file pone.0262301.s001.docx]

Stanford University School of Medicine Coronavirus Attitudes and Behaviors Survey

The Stanford University School of Medicine is interested in your experiences during the coronavirus pandemic. Your feedback is extremely important and will help policy-makers make accurate, real-time decisions that can help families and the economy.

The survey is voluntary and will take about 15 minutes to complete. Your answers will be kept confidential and will not be linked to your name. You may skip any questions that you do not want to answer.

If you have any questions or technical difficulties with the survey, please call the following toll free number 855-726-0790

If you agree to participate in this research, please select the ‘I Agree’ response to continue.

O I Agree

If respondent skips an item (left blank) the navigation continues to the next question unless specifically noted in the questions below.

---------------------------------

This survey asks about the impact of the coronavirus, also known as COVID-19, on your attitudes and behaviors.

1. About how long do you think the current public health crisis will continue?
   - Less than 2 weeks
   - At least 2 weeks but less than 1 month
   - At least 1 month but less than 3 months
   - At least 3 months but less than 5 months
   - 5 months or more

The next questions ask about how the coronavirus pandemic may have changed your daily life.

1. How has the time you spend at home changed since the pandemic?
   - Decreased a lot (by more than 50%)
   - Decreased somewhat (by less than 50%)
   - Has not changed
   - Increased somewhat (by less than 50%)
   - Increased a lot (by more than 50%)
   - I spent all of my time at home before the pandemic
2. How has the time you spend on each of the following activities changed since the pandemic? [will be shown as individual questions]

| Activity | **Decreased a lot**  (by more than 50%) | **Decreased somewhat**  (by less than 50%) | **Has not changed** | **Increased somewhat**  (by less than 50%) | **Increased a lot**  (by more than 50%) |  | **I didn’t do this before the pandemic** |
| --- | --- | --- | --- | --- | --- | --- | --- |
| Going to the grocery store | ⬜ | ⬜ | ⬜ | ⬜ | ⬜ |  | ⬜ |
| Receive personal services such as haircuts or manicures, or go to the gym | ⬜ | ⬜ | ⬜ | ⬜ | ⬜ |  | ⬜ |
| Exercise outdoors | ⬜ | ⬜ | ⬜ | ⬜ | ⬜ |  | ⬜ |
| Eat in a restaurant (not including take out or delivery) | ⬜ | ⬜ | ⬜ | ⬜ | ⬜ |  | ⬜ |
| Go to work outside your home | ⬜ | ⬜ | ⬜ | ⬜ | ⬜ |  | ⬜ |
| See a movie in a theater | ⬜ | ⬜ | ⬜ | ⬜ | ⬜ |  | ⬜ |
| Use shared transportation (such as commercial flights, trains, buses, or shared ride services) | ⬜ | ⬜ | ⬜ | ⬜ | ⬜ |  | ⬜ |

The next question asks about how your state or local area is responding to the coronavirus pandemic.

1. Are there directions from your governor or other officials to stay at home or shelter in place?
   - Yes [GO TO Q5]
   - No [GO TO Q6]
   - I don’t know [GO TO Q6 and skipped goes to Q6]

The next questions ask about what you would do if there were **not** directions to stay at home or shelter in place.

1. If there weren’t directions to stay at home, how would the time you spend on each of the following activities change? will be shown as individual questions

| Activity | **It would decrease a lot**  (by more than 50%) | **It would decrease somewhat**  (by less than 50%) | **It would not change** | **It would increase somewhat**  (by less than 50%) | **It would increase a lot**  (by more than 50%) |  | **I didn’t do this before the pandemic** |
| --- | --- | --- | --- | --- | --- | --- | --- |
| Going to the grocery store | ⬜ | ⬜ | ⬜ | ⬜ | ⬜ |  | ⬜ |
| Receive personal services such as haircuts or manicures, or go to the gym | ⬜ | ⬜ | ⬜ | ⬜ | ⬜ |  | ⬜ |
| Exercise outdoors | ⬜ | ⬜ | ⬜ | ⬜ | ⬜ |  | ⬜ |
| Eat in a restaurant (not including take out or delivery) | ⬜ | ⬜ | ⬜ | ⬜ | ⬜ |  | ⬜ |
| Go to work outside your home | ⬜ | ⬜ | ⬜ | ⬜ | ⬜ |  | ⬜ |
| See a movie in a theater | ⬜ | ⬜ | ⬜ | ⬜ | ⬜ |  | ⬜ |
| Use shared transportation (such as commercial flights, trains, buses, or shared ride services) | ⬜ | ⬜ | ⬜ | ⬜ | ⬜ |  | ⬜ |

<GO TO Q8>

<for those answering “no” or “I don’t know” OR BLANK to Q4>

The next questions ask about what you would do **if** there were directions to stay at home or shelter in place.

1. If you received directions from officials to stay at home, how would the time you spend on each of the following activities change?

**will be shown as individual questions**

| Activity | **It would decrease a lot**  (by more than 50%) | **It would decrease somewhat**  (by less than 50%) | **It would not change** | **It would increase somewhat**  (by less than 50%) | **It would increase a lot**  (by more than 50%) |  | **I didn’t do this before the pandemic** |
| --- | --- | --- | --- | --- | --- | --- | --- |
| Going to the grocery store | ⬜ | ⬜ | ⬜ | ⬜ | ⬜ |  | ⬜ |
| Receive personal services such as haircuts or manicures, or go to the gym | ⬜ | ⬜ | ⬜ | ⬜ | ⬜ |  | ⬜ |
| Exercise outdoors | ⬜ | ⬜ | ⬜ | ⬜ | ⬜ |  | ⬜ |
| Eat in a restaurant (not including take out or delivery) | ⬜ | ⬜ | ⬜ | ⬜ | ⬜ |  | ⬜ |
| Go to work outside your home | ⬜ | ⬜ | ⬜ | ⬜ | ⬜ |  | ⬜ |
| See a movie in a theater | ⬜ | ⬜ | ⬜ | ⬜ | ⬜ |  | ⬜ |
| Use shared transportation (such as commercial flights, trains, buses, or shared ride services) | ⬜ | ⬜ | ⬜ | ⬜ | ⬜ |  | ⬜ |

1. Think about the ways you have changed your behavior to limit the spread of the coronavirus.

On a scale of 1 to 10, how important was it to change your behavior to protect you and your family?

To answer, click on the line below, with 1 being not at all important and 10 being extremely important. If you need to change your answer, click again.

**1 10**
Not at all important Extremely important

1. Think about the ways you have changed your behavior to limit the spread of the coronavirus.

On a scale of 1 to 10, how important was it to change your behavior to protect other people?

To answer, click on the line below, with 1 being not at all important and 10 being extremely important. If you need to change your answer, click again.

**1 10**
Not at all important Extremely important

The next questions ask about the chances of you or someone like you catching the coronavirus.

Answer these questions with a number from 0 to 100, where "0" means that you think there is absolutely no chance, and "100" means that you think it is absolutely sure to happen.

For example, no one can ever be sure about tomorrow's weather, but if you think that rain is very unlikely tomorrow, you might say that there is a 10 percent chance of rain. If you think there is a very good chance that it will rain tomorrow, you might say that there is an 80 percent chance of rain.

For each of the following questions, click on the line below to show your answer. If you need to change your answer, click again.

1. How likely is it that you or someone like you would catch the coronavirus if you stayed at home the vast majority of the time?

**0 100**

1. How likely is it that you or someone like you would catch the coronavirus if you received personal services, such as haircuts or manicures, or went to the gym?

**0 100**

1. How likely is it that you or someone like you would catch the coronavirus if you exercised outdoors?

**0 100**

1. How likely is it that you or someone like you would catch the coronavirus if you went to the grocery store?

**0 100**

1. How likely is it that you or someone like you would catch the coronavirus if you ate at a restaurant, not including take out or delivery?

**0 100**

1. How likely is it that you or someone like you would catch the coronavirus if you went to work regularly outside your home?

**0 100**

1. How likely is it that you or someone like you would catch the coronavirus if you saw a movie in a theater?

**0 100**

1. How likely is it that you or someone like you would catch the coronavirus if you used shared transportation, such as commercial flights, trains, buses, or shared ride services?

**0 100**

The next questions ask about what would happen if you or somebody like you caught the coronavirus.

1. If you or somebody like you caught the coronavirus, how likely is it that you would not have any symptoms?

**0 100**

1. If you or somebody like you caught the coronavirus, how likely is it that you would need medical care?

**0 100**

1. If you or somebody like you caught the coronavirus, how likely is it that you would need to be hospitalized?

**0 100**

1. If you or somebody like you caught the coronavirus, how likely is it that you would die?

**0 100**

1. If you or somebody like you caught the coronavirus, how likely is it that the hospital would have the staff and supplies to treat you?

**0 100**

The next questions ask about coronavirus testing for you and your family.

1. Have you been tested by a health care provider for coronavirus?
   - Yes
   - No [GO TO 25 or Blank]
2. Was your test positive or negative?
   - Positive [GO TO Q26]
   - Negative
3. Do you currently have or have you had coronavirus symptoms?

   PROGRAMMING NOTE – ALLOW SELECTION OF BOTH YES OPTIONS.
   1. Yes – Currently have
   2. Yes – Had in the past
   3. No
4. Do you know if anyone in your family or among your close friends tested positive for coronavirus?
   - Yes
   - No

**26 A. OPEN #1. Currently, what worries you the most about the coronavirus pandemic?**

**26 B. OPEN #2. What, if anything, are you doing to deal with it?**

The coronavirus pandemic has caused financial challenges for many Americans. The next questions ask you about the financial impact on you and your household.

1. Compared to one month ago, how has your household income changed?
   - Decreased a lot (by more than 50%)
   - Decreased somewhat (by less than 50%)
   - Has not changed
   - Increased somewhat (by less than 50%)
   - Increased a lot (by more than 50%)
2. Compared to one month ago, how has your household’s typical weekly spending changed?
   - Decreased a lot (by more than 50%)
   - Decreased somewhat (by less than 50%)
   - Has not changed
   - Increased somewhat (by less than 50%)
   - Increased a lot (by more than 50%)
3. Overall, which one of the following best describes how well you are managing financially these days?
   - Finding it difficult to get by
   - Just getting by
   - Doing okay
   - Living comfortably
4. Please describe in your own words how you are managing financially during the pandemic.

1. Suppose that you have an emergency expense that costs $400. Based on your current financial situation, how would you pay for this expense?

|  | **Yes** | **No** |
| --- | --- | --- |
| Put it on my credit card and pay it off in full at the next statement | ⬜ | ⬜ |
| Put it on my credit card and pay it off over time | ⬜ | ⬜ |
| With the money currently in my checking/savings account or with cash | ⬜ | ⬜ |
| Using money from a bank loan or line of credit | ⬜ | ⬜ |
| By borrowing from a friend or family member | ⬜ | ⬜ |
| Using a payday loan, deposit advance, or overdraft | ⬜ | ⬜ |
| By selling something | ⬜ | ⬜ |
| Other (please specify) | ⬜ | ⬜ |
|  |  |  |
| I wouldn't be able to pay for the expense right now | ⬜ | ⬜ |

The next questions ask about you and your job.

1. Do you have a job that is deemed “essential,” such as a healthcare professional, police officer, firefighter, grocery store employee, or delivery personnel?
   - Yes
   - No
   - I’m not sure
2. Age

What is your age _____[ALLOW RANGE 18-110]

Don’t know _______

1. Gender

Are you …

Male

Female

Transexual/Transgender

Don’t Know

1. Are you of Hispanic, Latino, or Spanish origin?
   1. Yes
   2. No
   3. I’m not sure
2. What is your race? (Select all that apply.)
   1. White
   2. Black or African American
   3. American Indian or Alaska Native
   4. Asian
   5. Native Hawaiian or Other Pacific Islander
   6. Some Other Race (Please specify)
3. Marital status

Are you currently …

Married

Widowed

Divorced

Separated

Never Married

1. Including you, how many people are in your household?

____ [ALLOW RANGE 1-15]

1. What is the highest level of education that you have completed?

- Less than a high school diploma
- High school graduate or GED
- Some college or some technical school
- Associate's degree or professional certificate
- Bachelor's degree
- Master's or doctorate degree

1. What was your annual household income in 2019?
   - Less than $25,000
   - $25,000 to $49,999
   - $50,000 to $74,999
   - $75,000 to $149,999
   - $150,000 and over
   - Don’t know
   - Rather not answer
2. Has a health care professional ever diagnosed you with the following health conditions? [display as individual yes/no questions]
   - Hypertension or high blood pressure
   - Diabetes
   - Depression or anxiety
   - Heart disease
   - Respiratory diseases such as emphysema, asthma, chronic bronchitis, or chronic obstructive pulmonary disease (COPD)
   - Kidney disease
   - Autoimmune disorder such as rheumatoid arthritis or Crohn’s disease

We would like to send you $5 to thank you for completing this survey. If you would like to receive this, please provide your name. Your name and address will not be associated with your survey responses.

- Enter name: __________________-
- Submit survey without $5

We will send this in about 2 weeks to the address where we sent the letter you received.

Thank you. We appreciate your time and your help with this very important survey.

If you want more information about the COVID-19 pandemic or if you have been affected by the pandemic and are looking for resources, please visit the web sites below:

[www.cdc.gov](http://www.cdc.gov)

/coronavirus/2019-ncov/index.html

[www.dol.gov](http://www.dol.gov)

/coronavirus

Links Displayed as TEXT They are not active
