# Supplementary material for: Financial vulnerability and the impact of COVID-19 on American households
Source: PLoS One. 2022 Jan 14;17(1):e0262301. doi: 10.1371/journal.pone.0262301 (PMC8759691; doi:10.1371/journal.pone.0262301)
Supplement: Attachment — Submitted filename: Review PONE-D-21-15762.pdf [file pone.0262301.s002.pdf]

Review of PONE-D-21-15762

Financial vulnerability and the impact of COVID-19 on American households

Overall Assessment

Using U.S. survey data, the paper aims to examine the impact of the COVID-19 pandemic and associated depression in economic activity on consumers' level of financial vulnerability. While the topic of the paper is interesting, it fails to embed itself in existing literature on financial vulnerability that has provided validated measures of this variable using similar data that the authors use (e.g., Anderloni et al. 2012; Hoffmann and McNair 2019). Why do the authors not use one of these previously validated measures of financial vulnerability so that their article can join an ongoing discussion in the consumer studies literature and its results are able to be compared with such previous work? In general, I find the paper overly descriptive and a-theoretical. What do we know from the existing literature about how significant life events such as health shocks impact consumers' financial vulnerability and what new lessons do we learn here? Without answering these sort of questions, the paper leaves me wonder what new insights have been generated and what implications we can draw. More specific comments are the following:

- Page 3, lines 72-74: should it be "did NOT have emergency funds"? How else can you conclude that Black and Hispanic adults are more likely to experience financial hardship?
- Page 5, lines 108-109: it this gross or net annual income that you are referring to?
- Page 8, table 2: how does the COVID-19 infection rate of the sample compare to that of the general population?
- Page 11, table 4: many of the numbers such as for "Negative impact" do not add up to 100%. What am I missing here? Same for discussion on p. 12, lines 192-199.
- Page 14, table 6: it is not clear to me what kind of analysis you are reporting on here. Furthermore, why are you only classified as financially vulnerable if you score positive on 2 or more of the FV factors? Seems quite arbitrary. Why not 1 or more than 4?
- Page 16, lines 277-283: what does this tell us? Reader is left wondering what we have learned.

References

Anderloni, L., Bacchiocchi, E., and Vandone, D. (2012). Household Financial Vulnerability: An Empirical Analysis. *Research in Economics, 66*(3), 284-296.

Hoffmann, A. O. I., and McNair, S. J. (2019). How Does Consumers' Financial Vulnerability Relate to Positive and Negative Financial Outcomes? The Mediating Role of Individual Psychological Characteristics. *Journal of Consumer Affairs, 53*(4), 1630-1673.
