# Supplementary material for: Financial vulnerability and the impact of COVID-19 on American households
Source: PLoS One. 2022 Jan 14;17(1):e0262301. doi: 10.1371/journal.pone.0262301 (PMC8759691; doi:10.1371/journal.pone.0262301)
Supplement: Attachment — Submitted filename: Response to Reviewers.docx [file pone.0262301.s003.docx]

Response to Reviewers: The impact of COVID-19 on household finances

Reviewer #1

The reviewer’s point that our work does not include several previously-validated measures of financial vulnerability is well-taken. The paper is, generally, descriptive and a-historical. This is the result of the study design itself. We did not set out to examine financial vulnerability to COVID. Rather, we wanted to capture a snapshot of how Americans were responding to COVID in May 2020, along multiple axes of interest, including behavior, finances, and emotional well-being. As such, questions about finances were only one part of the questionnaire and included validated measures asking about changes in income and ability to cope with an unexpected expense. Our interest in financial vulnerability emerged upon analysis of the survey results, when we noted large disparities in the ability to cope financially with the pandemic reported by survey respondents. Hindsight being 20/20, we certainly wish we had included other measures of financial vulnerability, but we hope that our descriptive results may yet provide a fuller understanding of how some Americans experienced financial stress early in the pandemic. We have also added additional citations to the literature on financial vulnerability to further contextualize our results.

Page 3, lines 72-74: Corrected to “lacked” emergency funds

Page 5, lines 108-109: Specified that this referred to gross annual income

Page 8, table 2: Added footnote to clarify that the national incidence of COVID-19 among the U.S. adult population was 0.7, which falls within the 95% confidence interval of our sample.

Page 11, table 4: This was an error made by the corresponding author and has been fixed.

Page 14, table 6: Added text clarifying analysis and a footnote describing index more clearly

Page 16, lines 277-283: Added additional text to contextualize results and their implications

Reviewer #2

Line 145: Corrected

Line 255: Corrected spelling error

Lines 279-283: Added text to clarify results more

Lines 287-289: The table the text referred to was showing the incorrect figures; this was corrected.

Also added additional areas for research
