# Supplementary material for: Financial vulnerability and the impact of COVID-19 on American households
Source: PLoS One. 2022 Jan 14;17(1):e0262301. doi: 10.1371/journal.pone.0262301 (PMC8759691; doi:10.1371/journal.pone.0262301)
Supplement: Attachment — Submitted filename: Response to Reviewers.docx [file pone.0262301.s004.docx]

Response to Reviewers (2): The impact of COVID-19 on household finances

Reviewer #1

Thank you for the corrections on the references. These have been addressed.

Reviewer #3

Thank you for your comments. We agree that this is a descriptive analysis; although the response rate is in line with similar nationally-representative online surveys, we do want to be cautious in interpretation, as we cannot determine causality. We have added caveats and cautions about interpretation to our discussion.
